# Exploring Mental Health Issues in HCV-Positive Pregnant Women: A Qualitative Study

**DOI:** 10.1192/j.eurpsy.2024.1233

**Published:** 2024-08-27

**Authors:** A. Syed, S. S. Hashmi

**Affiliations:** ^1^First Faculty of Medicine, Charles University, Prague Czechia; ^2^Department Of Applied Psychology, National University Of Modern Languages, Karachi, Pakistan

## Abstract

**Introduction:**

The research was conducted to explore the mental health issues, including anxiety, depression, low mood, emotional irrationality, and stressors related to pregnancy in the tertiary care hospital, in Karachi, after getting diagnosed with a life-threatening virus of hepatitis C and to determine the factors associated with depression among HCV-infected pregnant women. There appears to be a dearth of literature on this particular topic, and depression in HCV-infected pregnant women might not be dealt with effectively till this gap in the literature is addressed. The findings are to aid counselors in the formulation of treatment plans to help the patients during pregnancy and it helped to address the gaps in the antenatal care plans and support provided to the vulnerable population like HCV-Infected pregnant women.

**Objectives:**

To explore what are the anxieties, stressors, and fears of HCV-infected young mothers.To explore the experiences of infected young mothers with HCV.

**Methods:**

We have used a qualitative design of the study and a convenient, purposeful sampling technique to acquire the data. In Karachi, Pakistan, a tertiary care hospital will host this trial. Young moms who registered HCV+ infections between January 2022 and June 2022 were included in the study. The tertiary healthcare setting was used for the investigation. The suggested number of 10 young moms with HCV who had been detected during pregnancy and came to the clinic for treatment were selected, those who provided consent and who were neither pregnant nor extremely unwell at the time of the study were eligible. The average age of the inhabitants was 26. There were 42.85% undergraduate mothers, 28.57% mothers with graduate degrees, and 28.57% mothers with postgraduate degrees in the population. Thematic analysis was utilized to evaluate the data, and the themes were generated by looking at the data and creating codes to look into the transcription’s content.

**Results:**

According to the findings, the referral system appeared to place a significant burden on individuals who were already dealing with the potentially fatal hepatitis C infection and were pregnant. In the antenatal period, when there should have been two different doctors’ visits, they were compelled to go to the same clinic.

**Conclusions:**

The finding addressed the importance of specialized care setting in the tertiary care hospital in Karachi, Pakistan. There is a requirement of training programs for the development of soft skills of health care professionals and there must be awareness sessions to promote and mobilize the understanding of the spread of this disease.

To this research finding the importance of comprehensive health care support was identified. And it also depicts the importance of inclusive antenatal program design.

**Disclosure of Interest:**

None Declared

